# Simultaneous Bilateral Quadriceps Tendon Rupture in an Adult Man

**DOI:** 10.5811/cpcem.2022.3.55675

**Published:** 2022-05-05

**Authors:** Marla C. Doehring, Steven L. Propst

**Affiliations:** * Indiana University, Department of Emergency Medicine, Indianapolis, Indiana; † University of Missouri, Department of Emergency Medicine, Springfield, Missouri

**Keywords:** case report, quadriceps tendon rupture, bilateral

## Abstract

**Case Presentation:**

A previously healthy 45-year-old man presented to the emergency department with bilateral knee pain and inability to extend his knees after a slip and fall on ice. The clinical diagnosis of bilateral quadriceps tendon rupture was confirmed by computed tomography (CT) of bilateral knees. The patient underwent successful operative repair the following day.

**Discussion:**

Bilateral quadriceps tendon rupture is rare and can be difficult to diagnose due to the impossibility of comparing the affected to the unaffected limb. Plain radiographs are usually not helpful, but ultrasound, CT, and magnetic resonance imaging may be used to confirm the clinical diagnosis.

## CASE PRESENTATION

A 45-year-old man with no significant past medical or surgical history presented to the emergency department after a mechanical fall. He took no daily medications and had a body mass index (BMI) of 44.2 kilograms per squared meter. The patient slipped on ice while walking and felt “pops” over both knees while falling. Exam demonstrated bilateral knees with no obvious swelling or bruising. He had good passive range of motion and mild tenderness to palpation superior to the patella on both knees. The patient was unable to perform straight leg raise with either leg.

**Image 1:**
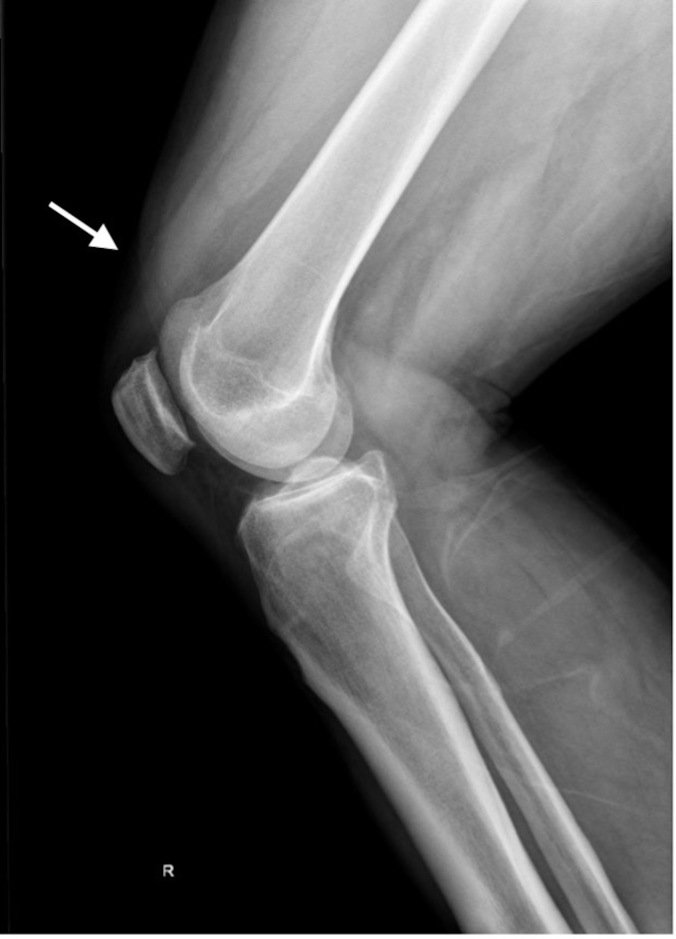
Plain radiograph of the right knee demonstrating mild suprapatellar soft tissue swelling (arrow).

**Image 2:**
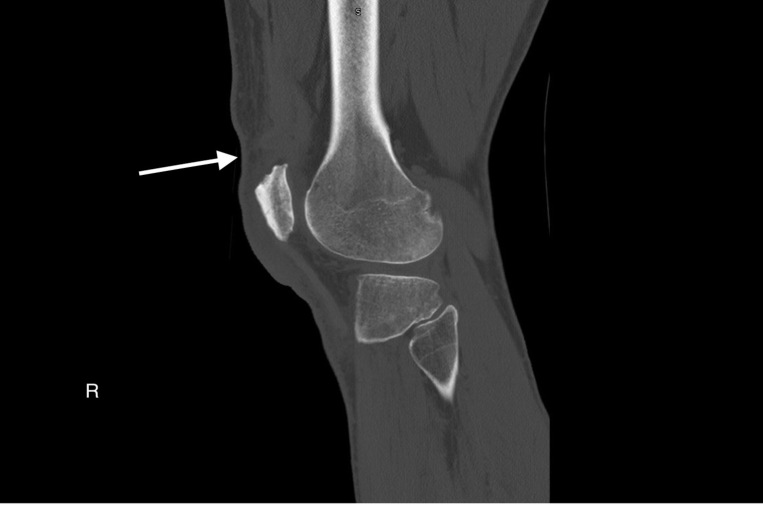
Computed tomography of right knee demonstrating near-complete tear of quadriceps tendon with retraction of the central portion of the tendon (arrow).

**Image 3:**
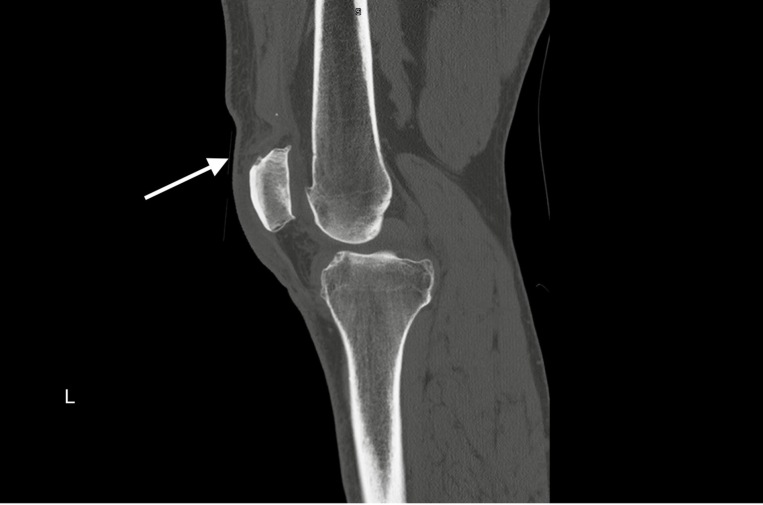
Computed tomography of left knee demonstrating near-complete tear of quadriceps tendon with retraction of the central portion of the tendon (arrow).

Plain radiographs of bilateral knees demonstrated mild soft tissue swelling over bilateral superior patellae but no other evidence of injury (Image 1). Computed tomography (CT) of bilateral knees confirmed the clinical diagnosis of simultaneous bilateral quadriceps tendon rupture (Images 2 and 3). Near-complete tears of both distal quadriceps tendons were surgically repaired the following day.

## DISCUSSION

Bilateral extensor mechanism rupture is rare and can be difficult to diagnose, with an initial missed rate reported up to 30–50%.^1^,^2^ Patients at risk usually have underlying comorbidities such as chronic renal disease, diabetes mellitus, rheumatologic disorder, chronic steroid use, or obesity. This patient had an elevated BMI but no other risk factors. The classic triad for clinical diagnosis of quadriceps tendon rupture is knee pain, inability to actively extend knee, and palpable suprapatellar gap.^2^,^3^

Tendon rupture often occurs due to indirect trauma. While attempting to regain balance the quadriceps rapidly contracts with the knee flexed. Rupture is likely from the maximum quadriceps contraction rather than the fall itself.^4^ The diagnosis is clinical, and plain films are rarely diagnostic. Ultrasound, CT, or magnetic resonance imaging may be useful to confirm the clinical diagnosis. In cases of bilateral quadriceps tendon rupture, the initial clinical diagnosis is made more challenging by the impossibility of comparing the affected limb to the unaffected limb. Treatment is surgical fixation followed by immobilization and physical therapy.^2^

**CPC-EM Capsule**

What do we already know about this clinical entity?

*Simultaneous bilateral quadriceps tendon rupture is a rare event, especially in patients without significant comorbidities*.

What is the major impact of the image(s)?

*Plain radiographs are rarely diagnostic but ultrasound, computed tomography, or magnetic resonance imaging may be helpful to make the diagnosis in the emergency department*.

How might this improve emergency medicine practice?

*Include bilateral quadriceps tendon rupture in the differential diagnosis when evaluating a patient whose clinical presentation is consistent with this rare entity*.
